# Plasticity of the inner cell mass in mouse blastocyst is restricted by the activity of FGF/MAPK pathway

**DOI:** 10.1038/s41598-017-15427-0

**Published:** 2017-11-09

**Authors:** M. Wigger, K. Kisielewska, K. Filimonow, B. Plusa, M. Maleszewski, A. Suwińska

**Affiliations:** 1 Department of Embryology, Faculty of Biology, University of Warsaw, Warsaw, Poland; 2 Faculty of Life Sciences, University of Manchester, Manchester, UK; 3 Present Address: Department of Experimental Embryology, Institute of Genetics and Animal Breeding, Polish Academy of Sciences, Jastrzębiec, Poland

## Abstract

In order to ensure successful development, cells of the early mammalian embryo must differentiate to either trophectoderm (TE) or inner cell mass (ICM), followed by epiblast (EPI) or primitive endoderm (PE) specification within the ICM. Here, we deciphered the mechanism that assures the correct order of these sequential cell fate decisions. We revealed that TE-deprived ICMs derived from 32-cell blastocysts are still able to reconstruct TE during *in vitro* culture, confirming totipotency of ICM cells at this stage. ICMs isolated from more advanced blastocysts no longer retain totipotency, failing to form TE and generating PE on their surface. We demonstrated that the transition from full potency to lineage priming is prevented by inhibition of the FGF/MAPK signalling pathway. Moreover, we found that after this first restriction step, ICM cells still retain fate flexibility, manifested by ability to convert their fate into an alternative lineage (PE towards EPI and vice versa), until peri-implantation stage.

## Introduction

Early mammalian development is characterised by a series of cell fate decisions, which lead to gradual restriction of developmental potential of the resultant cells. Changes in cell potency are accompanied by formation of primary cell lineages, first by setting apart the extraembryonic cell lineage – trophectoderm (TE) from the inner cell mass (ICM), followed by separation of pluripotent epiblast (EPI) and primitive endoderm (PE) within the ICM. PE emerges as a monolayer on the surface of the ICM and contributes to the endoderm layer of the extraembryonic yolk sac. PE identity results from the sequential activation of *Gata6*, *Pdgfrα*, *Sox17*, *Gata4* and *Sox7* genes^1–7^ and depends on the FGF/MAPK (Fibroblast Growth Factor/Mitogen-Activated Protein Kinase) signalling pathway^8–14^, which has also been shown to play a role in TE establishment^15^.

The pluripotent EPI, which expresses key pluripotency genes, including *Oct4*, *Sox2* and *Nanog* ^16–21^, gives rise to the body of the foetus. The timing of blastocyst lineage restriction has been widely investigated, but the results of these studies are contradictory and the ultimate answer has remained elusive. Embryonic stem cells (ESCs) are derived by culturing isolated ICMs^22–24^ and the efficiency of ESC derivation is dependent upon the precise blastocyst stage at which the ICM is isolated^22,25^. Thus, understanding the potency of cells within isolated ICMs and establishing the exact moment of irreversible cell fate commitment not only provides important insights into mouse embryogenesis in general, but also has a significant impact on optimisation of ESC derivation protocols.

The first cell fate decision, i.e. the establishment of the TE and ICM, was thought to depend on the position of cell within the embryo^26^ and apico-basal cell polarity^27^. During the last decade, further research has revealed that Hippo signalling is essential for the integration of information regarding cell-cell contact, cell polarisation and cell position, subsequently translating this information into cell lineage-specific transcriptional programmes^28^. The ICM was initially thought to comprise a homogeneous population of equivalent cells^29–31^. In line with this, formation of PE within the ICM was attributed to positional cues and ability of ICM cells to differentiate into this cell lineage in response to exposure to the outside environment. However, the finding that PE and EPI markers, Gata6 and Nanog, respectively, are expressed in an exclusive “salt and pepper” pattern already in E3.5 blastocysts evidenced the heterogeneity of ICM cells before formation of PE as an epithelium underlying the blastocyst cavity^2^. According to this model, ICM consists of a mixed population of EPI and PE precursors (pre-EPI and pre-PE cells)^2,7,19,32^ alongside bipotential cells co-expressing both markers^33^. The precursor and bipotential cells, which commit asynchronously to either fate, are segregated into respective layers, either inside or on the surface of the ICM^2,7,19,32^. These processes ensure the appropriate proportions of PE and EPI cells^33^. These novel findings have led to a paradigm shift in our understanding of lineage commitment and segregation in the mouse blastocyst and suggest that the mechanisms underlying developmental flexibility of ICM cells should be re-examined.

To gain insight into the process of ICM cells specification and to understand the mechanism that leads to ICM potency restriction we employed non-invasive time-lapse imaging and lineage tracing. We demonstrated that cells of TE-deprived ICMs maintain their totipotency, defined as an ability to differentiate into all primary cell lineages, only until 32-cell blastocyst stage (E3.0). The loss of totipotency is regulated by the activity of the FGF/MAPK signalling pathway and is accompanied by the formation of PE and EPI precursor cells within the ICM. From this stage onwards, isolated ICMs form only PE layer on their surface. However, flexibility of the ICM, i.e. ability of its cells to switch between PE and EPI fate, is maintained for a substantially longer time. In ICMs isolated from E3.5 embryos the ultimate PE layer originates from both presumptive EPI and PE precursors. At E4.5, however, the plasticity of isolated ICMs is highly reduced and the PE layer at this stage is formed mainly by proliferating PE cells originally present on the surface of the ICM, but in rare instances also by single EPI cells that convert their fate towards PE. The occurrence of cell conversion and cell death irrespective of cell position indicates that these mechanisms, apart from cell position and migration, act to preserve the balance between EPI and PE cells within the ICM of the blastocyst.

## Results

### Isolated E3.0 ICMs are able to reconstruct the TE epithelial layer

**Figure 1:**
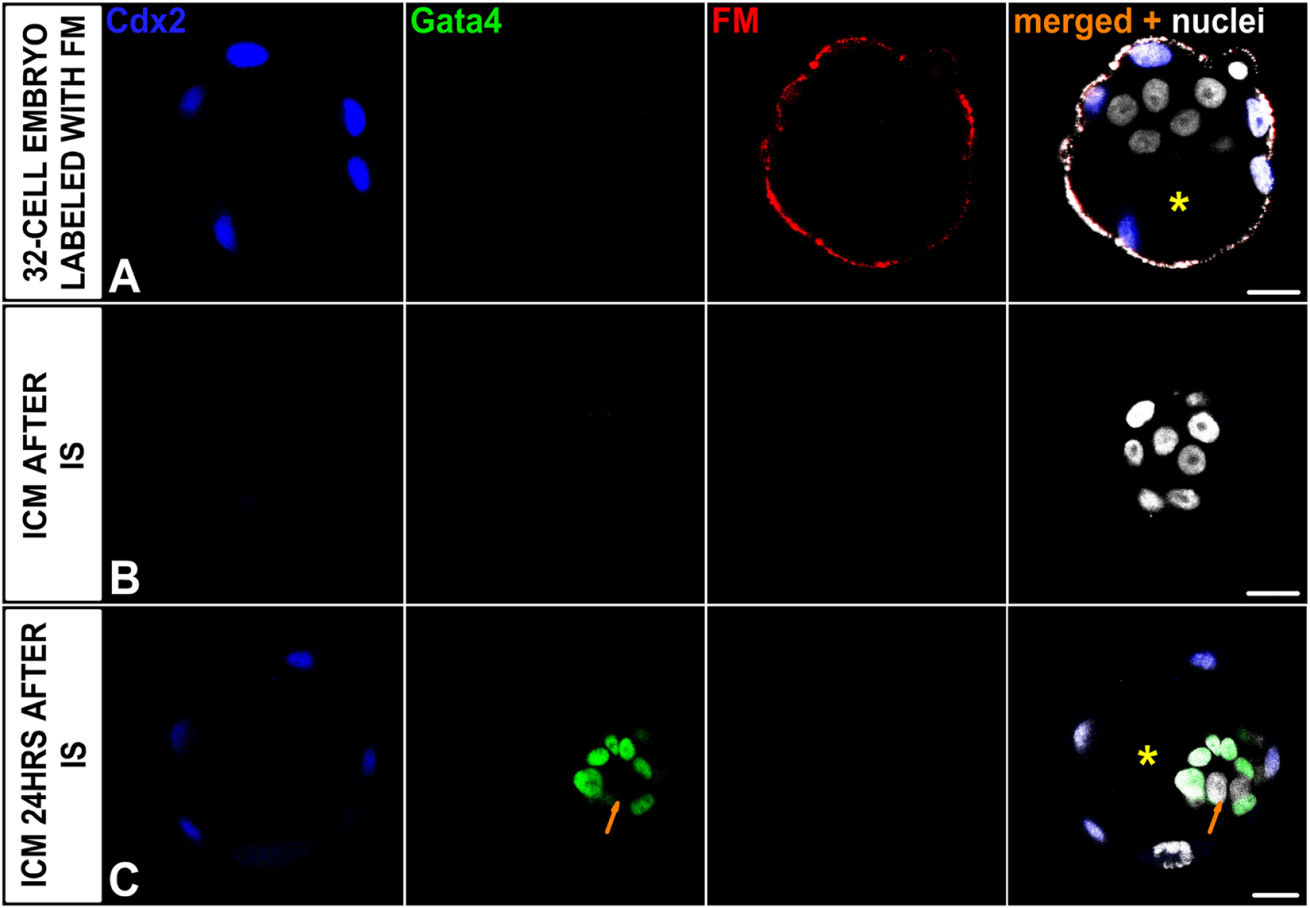
Control 32-cell blastocysts and their ICMs. **(A)** E3.0 blastocyst labelled with the fluorescent microspheres (FM), **(B)** E3.0 ICM immediately after isolation, **(C)** E3.0 ICM 24 hrs after IS. Blue: Cdx2, green: Gata4, red: FM labelling outer cells, white: nuclei; right panel shows merged pictures; yellow (*) indicates blastocyst cavity, orange arrow indicates group of presumptive EPI cells. Scale: 20 μm.

First, we examined whether ICM cells isolated from early E3.0 blastocysts are still totipotent and therefore able to recreate the TE layer. To exclude the possibility that the new TE layer is reconstituted from the original TE cells that survived immunosurgical (IS) isolation of the ICMs, we labelled the original TE layer of the blastocysts with fluorescent microspheres (FM) and then removed labelled cells by IS. In 15 intact, control E3.0 blastocysts, composed of 32.6 ± 3.3 cells, Cdx2 co-localised with FM proving that this marker was present only in cells of the outer TE layer (Fig. 1A). After immunosurgery, group of isolated ICMs was fixed to assess the efficiency of this procedure and confirm that they lack cells containing FM (i.e. TE cells). Fourteen out of 15 ICMs, fixed immediately after IS, did not contain any TE cells (Fig. 1B).

All 40 isolated ICMs derived from E3.0 blastocysts and cultured 24 hrs *in vitro* reconstituted blastocysts containing all cell lineages: Cdx2-positive TE cells surrounding the cavity as well as Gata4-positive PE cells and double-negative Gata4(−)/Cdx2(−) cells within the ICM (Fig. 1C). We confirmed that these double negative cells are indeed EPI cells by immunostaining additional 8 ICMs against EPI marker – Nanog together with the markers for TE and PE (Fig. S1). None of 48 cultured ICMs possessed FM-labelled cells, precluding the possibility that the newly formed TE layer originated from remaining TE cells that had not been removed by IS.

Taken together, these results indicate that at the 32-cell stage (E3.0) ICM cells are still totipotent and able to give rise to any primary cell lineage, including TE.

### Plasticity of the E3.0 single inner cells depends on the niche created by the surrounding cells

**Figure 2:**
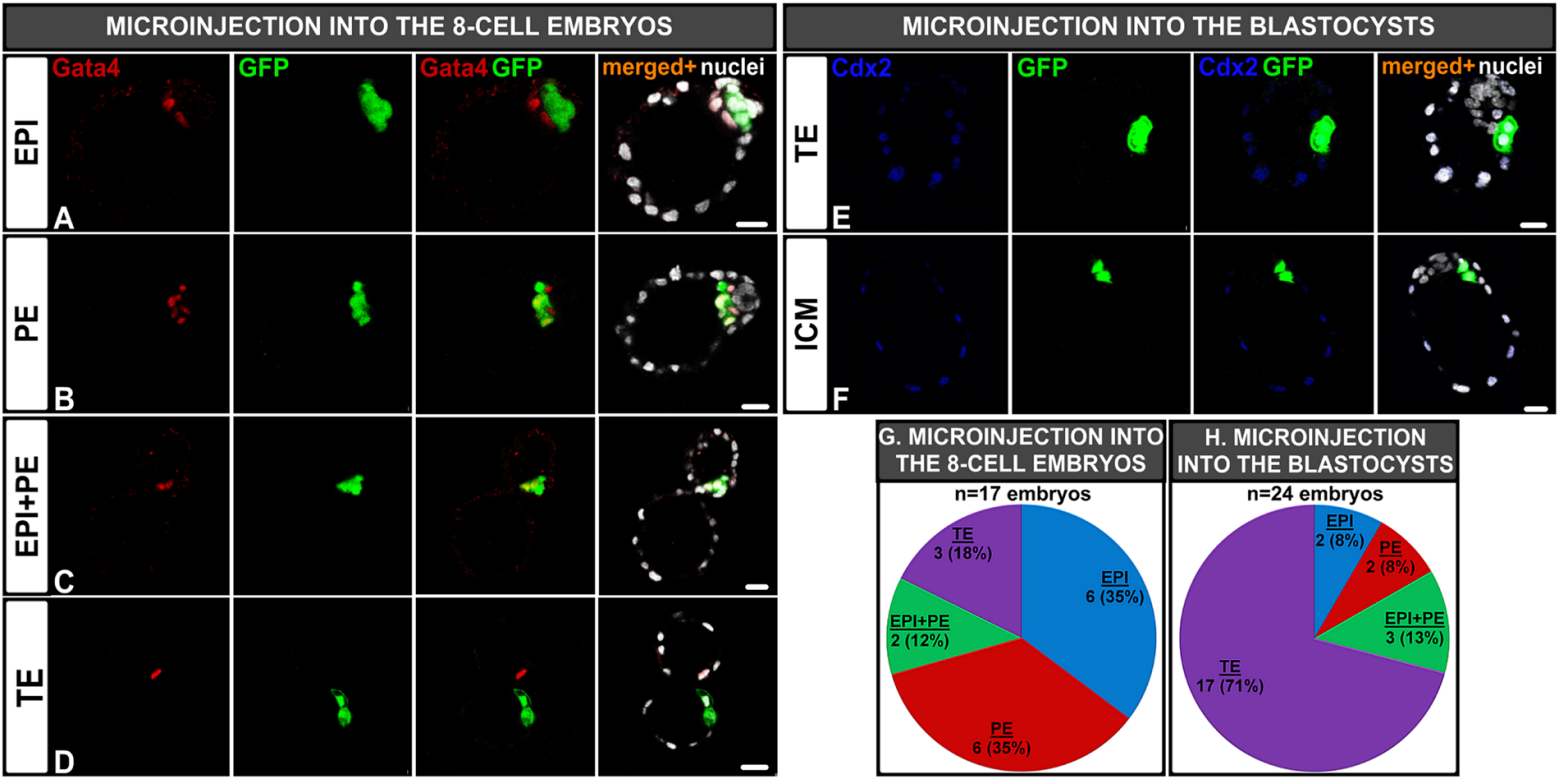
Localisation of the progeny of inner cells derived from E3.0 blastocyst introduced into the 8-cell embryo (**A**–**D** and **G**) and the blastocyst (**E**–**F** and **H**) after *in vitro* culture. Blastocyst with GFP-positive inner cells localised: **(A)** exclusively in EPI, (**B)** in PE, **(C)** both in EPI and PE, **(D)** in TE, **(E)** in TE (Cdx2 was up-regulated according to the new position), **(F)** exclusively in ICM. Red: Gata4, green: GFP, blue: Cdx2, white: nuclei; right panel shows merged pictures, **(G)** Lineage contribution of inner cells introduced into the 8-cell embryo after 72 hrs of culture, **(H)** Lineage contribution of inner cells introduced into the blastocyst after 48 hrs of culture. Scale: 20 μm.

To resolve whether the ability of ICM cells to differentiate into TE depends solely on their external position or/and microenvironment of the embryo, we microinjected single inner cells derived from E3.0 *GFP*-expressing embryos into the wild-type 8-cell embryos (Fig. 2A–D) or E3.5 blastocysts (Fig. 2E–F). Depending on the stage of the host embryo, the injected blastomere was located either in outer or inner position, respectively. When a single inner cell was introduced into the 8-cell embryo, its progeny was observed exclusively in either the EPI or PE lineage in the majority of the resulting blastocysts (6/17, 35%; Fig. 2A and G and 6/17, 35%; Fig. 2B and G, respectively). Two blastocysts (12%) had GFP-positive cells in both EPI and PE layers (Fig. 2C and G), while the remaining 3 blastocysts (18%) had progeny of the injected cell exclusively in the TE (Fig. 2D and G). Thus, despite the outer position, the injected blastomere progeny contributed to EPI and/or PE rather than TE. Surprisingly, when we microinjected 4 separated inner 32-cell stage blastomeres into the cavity of E3.5 blastocysts (to enforce their inner position), the vast majority of the recipient blastocysts (17/24, 71%) contained donor-derived cells only in TE (Fig. 2E and H). In the remaining 7 blastocysts (29%) *GFP*-expressing cells contributed to the ICM (Fig. 2F and H): either solely to EPI (2/24 blastocysts, 8%, Fig. 2H) or PE (2/24, 8%; Fig. 2H) or to both layers (3/24 blastocysts, 13%; Fig. 2H).

In summary, inner cells of 32-cell nascent blastocysts are in general totipotent, but their plasticity, i.e. their ability to differentiate into TE depends on the environment created by the surrounding cells, rather than solely on the outer position.

### Isolated E3.5 ICMs are no longer able to regenerate the TE but form the PE layer instead

Next we examined whether the ability of ICMs to differentiate into TE is maintained in E3.5 blastocysts, which already contain PE and EPI precursor cells. Control blastocysts (n = 22), which were fixed while the experimental blastocysts were subjected to IS, had an average of 51.0 ± 8.2 cells, with 33 ± 3.7 cells in the TE and 11.6 ± 2.3 cells in the ICM. Gata4-positive (8.0 ± 1.9) and Gata4-negative (3.6 ± 1.1) cells that localised within the ICM have not yet been sorted at this stage, as was previously reported^2,7,18^.

**Figure 3:**
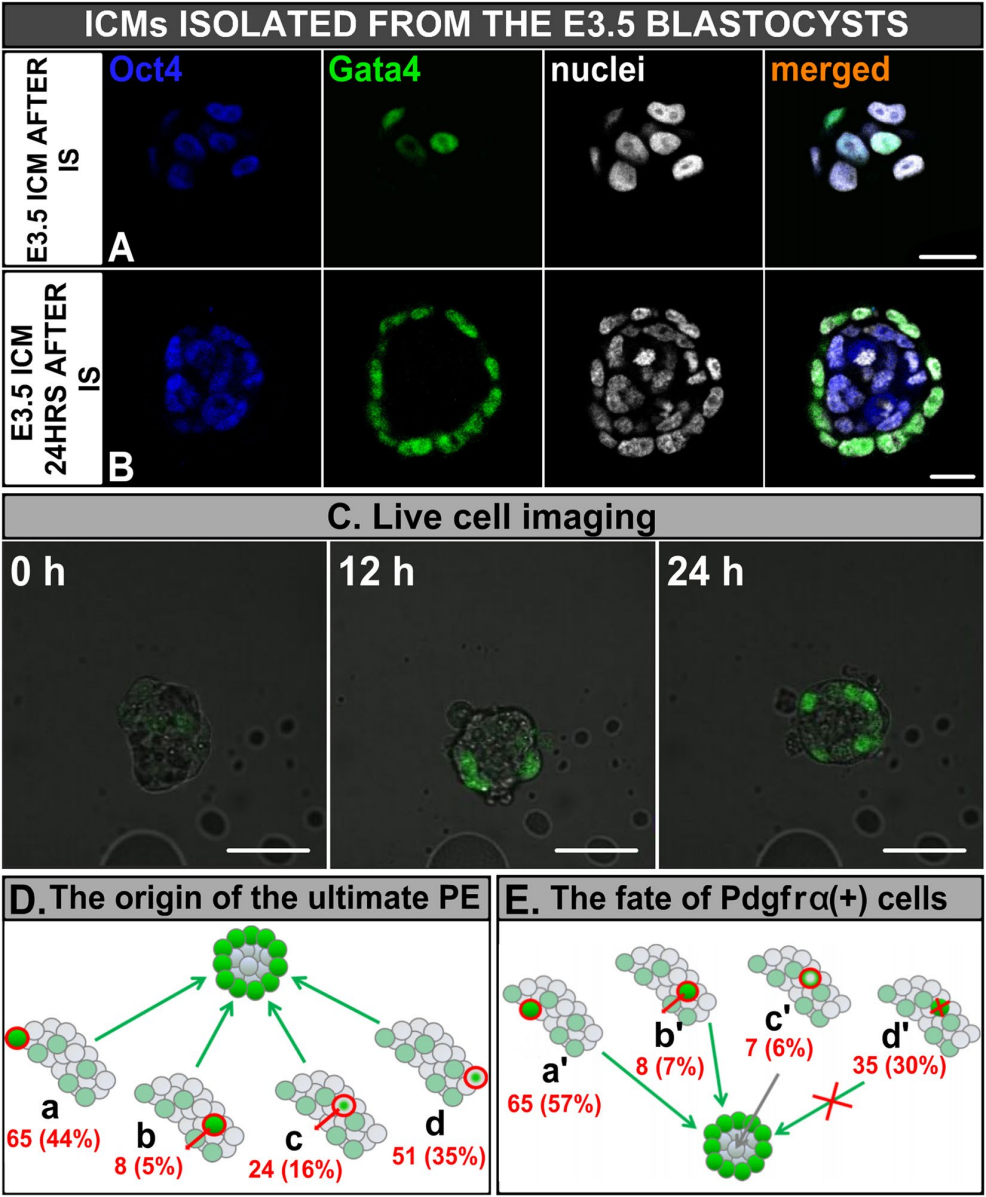
Development of E3.5 ICMs. **(A)** ICM immediately after IS, and **(B)** 24 hrs after IS. Blue: Oct4, green: Gata4, white: nuclei; right panel shows merged pictures, **(C)** Time-lapse imaging of E3.5 ICM immediately after isolation, after 12 hrs and after 24 hrs of *in vitro* culture (single optical sections), **(D)** The scheme of the ultimate PE origin: (a) *Pdgfrα* ^*H2B-GFP*^-expressing cells localised on the surface of isolated ICM from the beginning of *in vitro* culture, (b) *Pdgfrα* ^*H2B-GFP*^-expressing cells which translocated from inside to outside, (c) Pdgfrα^H2B-GFP^-negative cells which up-regulated *Pdgfrα* ^*H2B-GFP*^ during *in vitro* culture and migrated outside, (d) Pdgfrα^H2B-GFP^-negative cells which up-regulated *Pdgfrα* on the surface of ICM and maintained this position, **(E)** The scheme of the fate of all *Pdgfrα* ^*H2B-GFP*^-expressing cells: (a’) cells which were localised on the surface of ICM, contributing to PE, (b’) cells which were initially placed inside and translocated outside, contributing to PE, (c’) cells localised inside, which down-regulated *Pdgfrα* ^*H2B-GFP*^ during culture, (d’) cells which underwent apoptosis. Scale: 20 μm.

First, we investigated whether ICMs freshly isolated from E3.5 blastocysts (Fig. 3A) and subsequently cultured *in vitro*, are able to regenerate TE, thus recreating a blastocyst-like structure. We analysed 22 E3.5 ICMs altogether and found that although in 64% (7/11) of ICMs isolated from E3.5 blastocysts and cultured for 24 hrs and in 27% (3/11) of ICMs cultured for 48 hrs single Cdx2-positive TE cells avoided lysis during IS and remained on their surface, they were unable to recreate the TE layer. Moreover, during *in vitro* culture the number of TE cells did not increase significantly and the number of ICMs containing TE decreased, suggesting that the TE cells, which survived the IS procedure, failed to proliferate and probably ultimately underwent lysis.

After 24 hrs of *in vitro* culture E3.5 ICMs (n = 11) formed a layer of Gata4-positive PE cells that completely enveloped inner *Oct4*-expressing EPI cells (Fig. 3B). The mean number of ICM cells was 38.2 ± 6.9 with 23.4 ± 5.7 Gata4-positive PE and 13.6 ± 2.8 Oct4-positive EPI cells, which is similar to cell numbers observed in ICMs in control blastocysts cultured for 24 hrs (n = 9; total cell number 35.6 ± 10.8, with 19.9 ± 8.8 PE and 15.7 ± 10.4 EPI cells; p > 0.05). After 48 hrs of culture the number of cells in isolated ICMs (n = 11) increased to 102 ± 27.6 (76.0 ± 19.74 PE and 23.7 ± 15.5 EPI cells) and showed an increased proportion of PE to EPI cells (3.2:1 at 48 hrs *vs*. 1.7:1 at 24 hrs), which might reflect a difference in proliferation rate, apoptosis or reprogramming (cell numbers increased by 3.25 fold for PE *vs*. 1.74 fold for EPI). In these isolated ICMs cultured for 48 hrs, PE cells formed a multilayered envelope surrounding the core of Gata4(−) and Cdx2(−) presumptive EPI cells.

In summary, ICMs isolated from E3.5 blastocysts create PE on their surface and fail to form TE, indicating that at this stage presumptive EPI and PE precursors are not able to differentiate into TE.

### The PE formed on the surface of E3.5 ICMs originates from both Pdgfrα^H2B-GFP^ –positive and Pdgfrα^H2B-GFP^ –negative cells

In order to determine the origin of PE cells forming the surface layer of isolated E3.5 ICMs, we subjected such ICMs to time-lapse recording for 24 hrs (n = 17). In this experiment we used blastocysts derived from Pdgfrα^H2B-GFP^ reporter mice^34^. As Pdgfrα is a faithful marker of PE cells at this stage of embryonic development^7,35^, we were able to examine whether the final PE layer was derived exclusively from *Pdgfrα* ^*H2B-GFP*^-expressing cells or from both Pdgfrα^H2B-GFP^(+) and Pdgfrα^H2B-GFP^(−) cells, which changed their fate after removal of the TE. To confirm the reliability of Pdgfrα^H2B-GFP^ marker we fixed and immunostained all ICMs at the end of *in vitro* culture for Gata4 and proved that both proteins co-localise in PE cells (Fig. S2A).

We observed that cultured ICMs established an outer layer of *Pdgfrα* ^*H2B-GFP*^-expressing cells and that the GFP signal intensity within these cells was gradually enhanced during culture (Movie S1, Fig. 3C, Table S1). The PE layer on the surface of isolated ICMs (148 cells in 17 embryos) after 24 hrs of culture was derived from 4 distinct types of cells (Fig. 3D). The first group consisted of Pdgfrα^H2B-GFP^(+) cells, which were localised on the surface of isolated ICMs from the beginning of the *in vitro* culture (Fig. 3D-a). Progeny of these cells constituted 44% of the final PE layer (65/148 cells). The second group (8/148 cells; 5%) included progeny of cells that localised inside the ICM at the time of ICM isolation and migrated to the outside layer during the culture, while showing *Pdgfrα* ^*H2B-GFP*^ expression throughout the culture (Fig. 3D-b). The remaining cells that constitute the ultimate PE originated from cells that did not express *Pdgfrα* ^*H2B-GFP*^ initially, but up-regulated its expression during *in vitro* culture, suggesting that they acquired PE fate during culture. 16% of the final PE cells (24/148) were progeny of cells that up-regulated *Pdgfrα* ^*H2B-GFP*^ inside the ICM and then migrated to the surface (Fig. 3D-c), and 35% (51/148) – came from cells that up-regulated *Pdgfrα* ^*H2B-GFP*^ while localised on the surface of the ICM throughout the culture period (Fig. 3D-d).

Overall, we found that ICMs isolated from E3.5 blastocysts become surrounded by a PE layer that originates from both Pdgfrα^H2B-GFP^-positive and Pdgfrα^H2B-GFP^-negative cells, capable of modulating their fate at this stage. As Pdgfrα^H2B-GFP^-negative cells that eventually contributed to the PE layer were localised both on the surface and inside the ICM at the start of the culture, formation of the PE could not have been induced solely by exposure to the external environment.

### *Pdgfrα*^*H2B-GFP*^-expressing cells present in the E3.5 blastocyst may contribute to both PE and EPI lineages within the isolated ICM

We also analysed the fate of cells expressing *Pdgfrα* ^*H2B-GFP*^ at the beginning of the culture (Fig. 3E, Table S2). We found that more than half of those cells (57%, 65/115) were initially localised on the surface of the isolated ICM and contributed to the ultimate PE (Fig. 3E, group a’). Only 7% (8/115 cells) was present in deeper layers of the ICM, and moved to the surface during the culture, becoming a part of the final PE (Fig. 3E-b’). Seven out of 115 cells (6%) initially expressing *Pdgfrα* ^*H2B-GFP*^ were located inside the ICM at the time of its isolation, but down-regulated *Pdgfrα* ^*H2B-GFP*^ expression, during *in vitro* culture (Fig. 3E-c’). The remaining 35 Pdgfrα^H2B-GFP^-positive cells (30%) underwent cell death (Fig. 3E-d’). Cell death involved not only pre-PE cells that originally localised inside the ICM, but also pre-PE cells present on the surface of the ICM just after isolation (59 and 41% of all pre-PE cells undergoing cell death, respectively).

In summary, some cells, despite expression of PE-specific marker, remain flexible and able to convert into EPI cells. Thus, presumably down-regulation of *Pdgfrα*, apart from position, migration and apoptosis, contributes to regulation of the final number and proportions of cells building the ICM.

### E4.5 ICMs do not regenerate the TE and form the PE layer by cell spreading and migration

**Figure 4:**
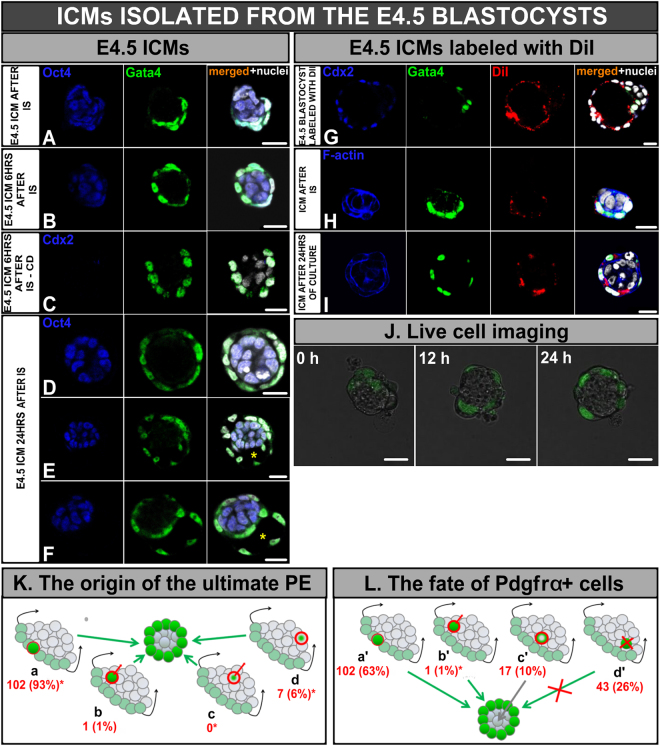
Development of E4.5 ICMs. **(A)** ICM immediately after IS, and **(B)** 6 hrs after IS, **(C)** ICM treated with cytochalasin D (CD) 6 hrs after IS, **(D**–**F)** ICMs 24 hrs after IS: (**D**) surrounded by single layer of PE, **(E**) containing cavity, (**F**) surrounded by two layers of PE separated by cavity, **(G)** E4.5 blastocyst labelled with DiI dye, **(H)** E4.5 ICMs with DiI-marked PE layer immediately after IS and **(I)** 24 hrs after IS. Blue: Oct4 or Cdx2 or F-actin, green: Gata4, red: DiI, white: nuclei_;_ right panel shows merged pictures; yellow (*) indicates blastocyst cavity, **(J)** Time-lapse imaging of E4.5 ICM immediately after isolation, after 12 hrs and after 24 hrs of *in vitro* culture (single optical sections), (**K**) The scheme of the ultimate PE origin: (a) *Pdgfrα* ^*H2B-GFP*^-expressing cells, which maintained their outside position, (b) *Pdgfrα* ^*H2B-GFP*^-expressing cells, which translocated from inside to outside, (c) Pdgfrα^H2B-GFP^-negative cells, which up-regulated *Pdgfrα* ^*H2B-GFP*^ expression during culture, (d) placed outside Pdgfrα^H2B-GFP^-negative cells, which up-regulated *Pdgfrα* on the surface of ICM, **(L)** The scheme of the fate of all *Pdgfrα* ^*H2B-GFP*^-expressing cells: (a’) cells which were localised on the surface of ICM, (b’) cells which changed their localisation from inside to outside, (c’) cells which down-regulated *Pdgfrα* ^*H2B-GFP*^, (d’) cells which underwent apoptosis. (*) statistically significant difference compared to corresponding cell groups of E3.5 ICMs (p < 0.05). Scale: 20 μm.

Next, we examined the development of isolated ICMs derived from E4.5 blastocysts. The mean number of cells in control blastocysts was 115.9 ± 19.3 (n = 7), of which 87.3 ± 14.7 cells constituted the TE, while the remaining cells formed the ICM, which consisted of 16.9 ± 6.9 PE and 11.7 ± 1.9 EPI cells. Immediately after removal of TE, ICMs isolated from E4.5 blastocysts (n = 8) contained similar numbers of cells as ICMs in intact control blastocysts (25.6 ± 7.5 with 13.8 ± 4.9 PE cells and 11.1 ± 3.8 EPI cells; p > 0.05, Fig. 4A).

Using immunostaining, we first checked the ability of E4.5 ICMs to regenerate TE. Similarly to ICMs from E3.5 blastocysts, E4.5 ICMs (n = 32) were no longer able to recreate blastocyst-like structures containing Cdx2-positive TE. Instead, a layer of Gata4-postive cells encompassing inner Oct4-positive EPI cells was formed (Fig. 4B). In order to examine the dynamics of PE layer formation, we fixed ICMs 6 hrs after IS (n = 24). The total number of ICM cells (28.1 ± 11.1) and the number of cells belonging to PE and EPI (16.6 ± 6.5 and 11.3 ± 4.1 respectively) did not increase compared to the numbers of the respective cells in ICMs immediately after isolation (n = 8) (25.7 ± 7.5 *vs*. 28.1 ± 11.1; p > 0.05), indicating that the PE layer was formed by flattening and extension of existing PE cells rather than from proliferation of these cells or reprogramming of EPI cells.

To verify this hypothesis, we subjected freshly isolated ICMs to cytochalasin D (CD) – an agent that causes the depolymerisation of actin microfilaments and thus inhibits cell migration and cytokinesis. In contrast to all control ICMs (n = 24), which became entirely surrounded by the extended PE cells within 6 hrs of culture, 7 out of 8 CD-treated ICMs were only partially covered by round, loosely arranged PE cells (Fig. 4C). Moreover, the number of PE cells in CD-treated and control ICMs were similar (16.9 ± 7.9 and 16.4 ± 4.8, respectively; p > 0.05). These results confirm that progressive covering the cultured ICMs by PE cells is the result of migration and extension of the PE shortly after removal of the TE.

After 24 hrs of culture in standard medium, isolated ICMs became entirely surrounded by PE cells. The mean number of ICM cells increased almost two fold and reached 61.9 ± 15.8 (n = 32). Each ICM was assigned to one of three morphological categories: ICMs that formed a monolayer of PE on their surface (14/32, 44%, Fig. 4D), ICMs covered by a monolayer of PE cells and containing a cavity (12/32, 38%, Fig. 4E), and ICMs that formed structures resembling egg cylinders with PE split into two layers separated by a cavity, supposedly mimicking visceral and parietal endoderm (6/32, 19%, Fig. 4F).

### The PE layer on the surface of E4.5 ICMs after *in vitro* culture originates mainly from PE cells originally localised on the ICM surface

To confirm whether the PE layer in E4.5 cultured ICMs is derived from PE cells originally present on the surface of the ICM or from EPI cells which still retain the capacity to convert their fate, we labelled PE cells present on the ICM surface by microinjecting a lipophilic fluorescent dye (DiI) into the cavity of E4.5 blastocysts (Fig. 4G), which were then subjected to IS to remove the TE cells. Isolated ICMs with a labelled PE layer were cultured for 24 hrs and then subjected to immunostaining for Gata4. We observed Gata4 staining in 99% of DiI-labelled cells (11.9 ± 4.7/12.0 ± 4.6) in control ICMs (n = 8) which were fixed immediately after labelling, thus confirming the efficiency of our labeling method (Fig. 4H).

All of the isolated DiI-labelled ICMs (n = 37) were completely surrounded by a PE layer after 24 hrs of culture. The final PE layer was composed of 27.11 ± 7.7 cells, of which 24.97 ± 7.1 cells (92%) also showed staining for Gata4 (Fig. 4I), suggesting that the main source of the PE layer were PE cells present on the ICM surface at the moment of its isolation. The remaining 2.14 ± 3.2 (8%) Gata4-positive cells lacked labelling, which could have resulted either from dilution of the dye following cell division, or the ability of EPI cells to convert towards PE fate. Moreover, in 12 out of 37 ICMs single Gata4-negative EPI cells (0.92 ± 1.7/14.03 ± 6.6, i.e. 7%) were labelled with dye. These DiI-containing EPI cells originated either from PE cells still able to change their fate, or from unsorted EPI cells, which at the time of labelling occupied the surface position, and thereby became dye-labelled.

To dissect the cellular origin of the final PE layer we applied time-lapse imaging to E4.5 ICMs isolated from blastocysts expressing *Pdgfrα* ^*H2B-GFP*^ (n = 9). In contrast to ICMs isolated from E3.5 blastocysts, the intensity of GFP fluorescence (Pdgfrα^H2B-GFP^) was strong from the onset of imaging and did not show a noticeable increase during culture (Fig. 4J, Fig. S2B).

Time-lapse recordings allowed us to distinguish 3 groups of cells that contributed to the final PE layer (n = 110 cells in 9 embryos; Fig. 4K, Table S1). The vast majority of the ultimate PE cells (102/110 cells, 93%) was derived from *Pdgfrα* ^*H2B-GFP*^-expressing cells, which were positioned on the surface of the ICM from the beginning and did not change their peripheral positioning during culture (Fig. 4K, group a). The percentage of such cells in E4.5 ICMs was double that observed in E3.5 ICMs (93% *vs*. 44%, p < 0.05). Pdgfrα^H2B-GFP^-positive cells, which were initially located in the interior of the ICM and migrated towards the surface as the culture progressed, accounted for only 1% (1/110 cells; Fig. 4K-b). A small number of Pdgfrα^H2B-GFP^-negative cells positioned on the outside (7/110) started to express *Pdgfrα* ^*H2B-GFP*^ during imaging, suggesting conversion of EPI to PE was taking place (Fig. 4K-d). Such conversion was less frequent when compared to ICMs isolated from E3.5 blastocysts (6% *vs*. 35%, respectively, p < 0.05). In contrast to E3.5 ICMs, E4.5 ICMs did not contain EPI cells that were originally localised inside the ICM, converted their fate to PE, and migrated to the surface, thus reinforcing the PE layer (0 *vs*. 16%).

### PE cells present in the isolated E4.5 ICM can still convert into EPI cells

To determine whether cells of E4.5 ICMs are still able to switch between PE and EPI fates, we also analysed the destiny of Pdgfrα^H2B-GFP^-positive cells at the start of the imaging period (n = 163 cells in 9 embryos) (Fig. 4L; Movie S2, Table S2). At this initial timepoint, 63% of these cells (102/163) were located on the surfaces of the ICMs throughout the whole culture (Fig. 4L, group a’). The remaining 37% Pdgfrα^H2B-GFP^–positive cells were localised inside the ICM. We observed only 1 cell (1%) that changed its localisation from inside to outside and ultimately contributed to the PE layer (Fig. 4L-b’), whereas 10% of cells (17/163) underwent conversion to EPI (Fig. 4L-c’) and remained inside, and 26% of cells (43/163) underwent cell death (Fig. 4L-d’). The percentage of Pdgfrα^H2B-GFP^–positive cells that were localised inside the E4.5 ICM and moved outside during culture decreased significantly in comparison to E3.5 ICMs (1% *vs*. 7%.; p < 0.05).

Taken together, our results demonstrate that the PE layer which covers the surface of isolated E4.5 ICMs after culture originates predominantly from existing segregated PE cells that are present on the ICM surface already at the start of *in vitro* culture. However, there are still some ICM cells that retain the ability to change their fate, as reflected by up- or down-regulation of *Pdgfrα* ^*H2B-GFP*^ expression. This clearly shows that although the fate of most ICM cells at the peri-implantation blastocyst stage is restricted, some cells still retain plasticity.

### Cells in isolated E3.5 ICMs retain full potency upon FGF4/MAPK inhibition

We noticed that ICMs lost their ability to differentiate into TE around E3.5, i.e. at the moment when PE and EPI precursors expressing lineage-specific markers first appear. It has been suggested that activity of the FGF4/MAPK signalling pathway is required for the switch from an overlapping to exclusive expression of Nanog and Gata6 markers, characteristic for EPI and PE progenitors, respectively^8^. We therefore asked whether modulation of FGF pathway activity influences the developmental plasticity of ICM cells.

**Figure 5:**
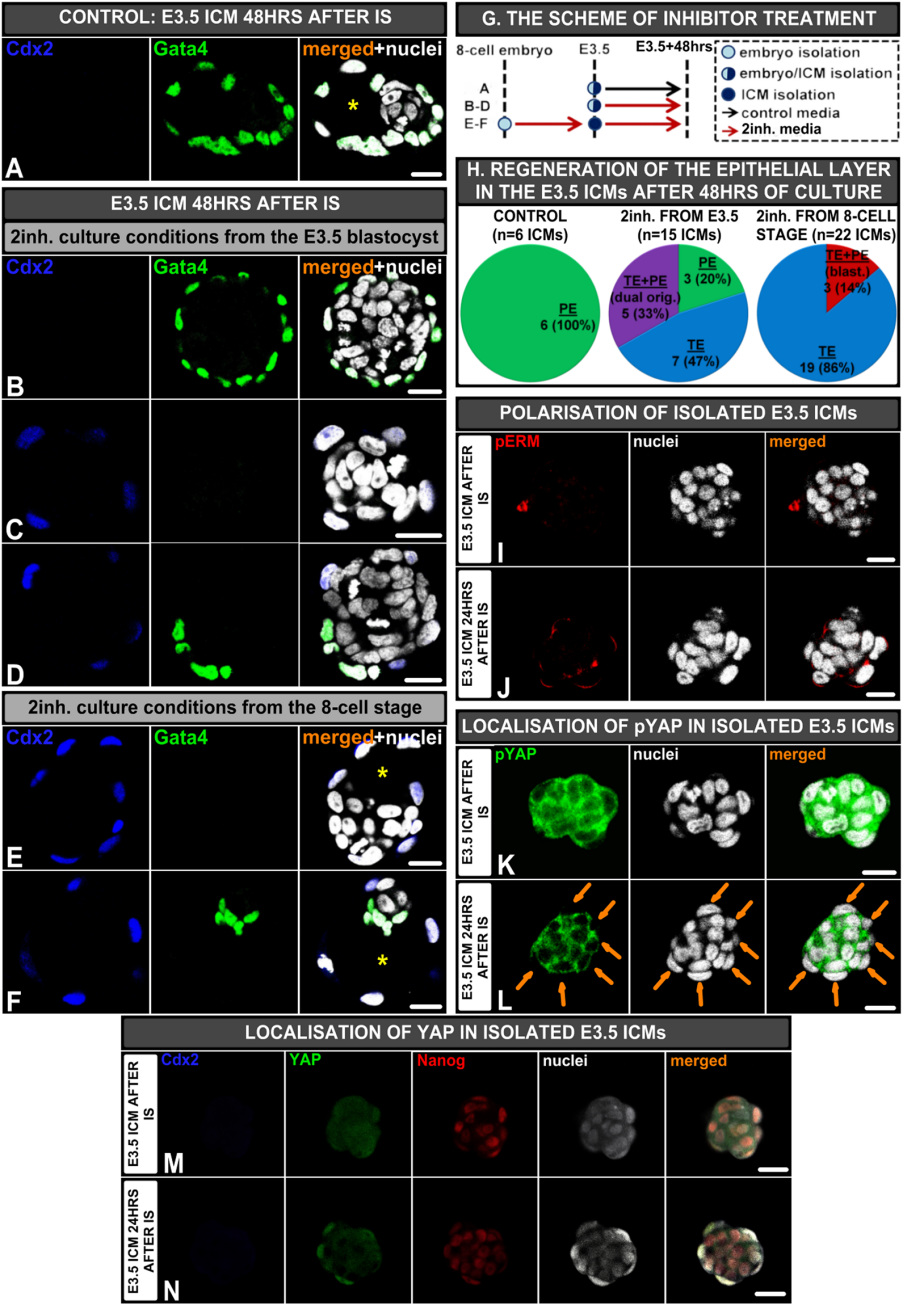
Effect of FGF/MAPK inhibition on isolated E3.5 ICMs. **(A)** Control E3.5 ICM cultured 48 hrs, **(B**–**D)** ICMs derived from E3.5 blastocysts and afterwards cultured 48 hr hrs in 2inh: **(B**) ICM with the outer PE layer, **(C)** ICM with the outer TE layer, and **(D)** ICM with outer heterogeneous (PE and TE) layer, **(E**,**F)** ICMs derived from E3.5 blastocysts pre-incubated in 2inh. media from the 8-cell stage: **(E)** ICM with the outer TE layer, **(F)** Blastocyst with the TE, EPI and PE, **(G)** The time schedule of inhibitor treatment; red and black arrows indicate the culture periods in the presence or absence of inhibitors, respectively, **(H)** Composition of epithelial layer in isolated ICMs cultured 48 hrs, **(I**,**J)** Polarisation of E3.5 ICMs (isolated from embryos pre-incubated in 2inh. conditions from the 8-cell stage) immediately **(I)** and 24 hrs after IS **(J)**, **(K**,**L)** Localisation of pYAP in E3.5 ICMs (isolated from embryos pre-incubated in 2inh. conditions from the 8-cell stage) immediately **(K)** and 24 hrs after IS **(L)**, **(M**,**N)** Localisation of nuclear YAP in E3.5 ICMs immediately **(M)** and 24 hrs after IS **(N)**; yellow (*) indicates blastocyst cavity, orange arrows indicate ICM cells devoid of pYAP signal. Scale: 20 μm.

To test this hypothesis, we first inhibited FGF4/MAPK signalling pathway by treatment of E3.5 ICMs with FGFR2/MEK inhibitors (2inh.)^12^ for 48 hrs. As expected, control ICMs, cultured in a standard medium, formed PE on their surface (n = 6; Fig. 5A,G and H). In contrast, among 15 ICMs cultured in 2inh. conditions only 3 formed a Gata4-positive PE layer (20%, Fig. 5B,G and H), whereas 7 regenerated a Cdx-2-positive TE layer (47%; Fig. 5C,G and H) and 5 generated a continuous epithelial layer of dual-origin, containing TE and PE cells (33%; Fig. 5D,G and H).

Bearing in mind that from E3.25 to E4.5 reversibility is possible after removal of inhibitors^36^ and to ensure that we inhibit FGF signalling efficiently, in a separate experiment we pre-incubated the embryos in the presence of FGFR2/MEK inhibitors from the 8-cell stage until formation of E3.5 blastocyst. Blastocysts were then subjected to IS, followed by continued culture in 2inh. conditions for 24 or 48 hrs. Along with ICMs subjected to 2inh. treatment, intact blastocysts were cultured in the same conditions to verify whether inhibition of PE formation was efficient. We confirmed that all blastocysts developed from the 8-cell stage in 2inh. conditions contained ICMs completely lacking PE cells (n = 13; Fig. S3), while control blastocysts displayed typical pattern of cell lineage markers with *Sox2*-expressing EPI cells and *Sox17*-expressing PE cells (n = 7; Fig. S3).

We found that control ICMs, pre-incubated from the 8-cell stage and cultured after isolation in standard medium for 24 hrs (n = 15) or 48 hrs (n = 7), formed the PE layer. In contrast, all ICMs that developed from the blastocysts pre-incubated in the 2inh. medium from the 8-cell stage and cultured for 48 hrs (n = 22) regenerated a layer of Cdx-2-positive outer TE cells (Fig. 5E,G and H) surrounding Nanog-positive EPI cells (Fig. S4). Additionally, in 3 cases ICMs created blastocysts composed of EPI cells and Gata4-expressing PE cells (Fig. 5F,G and H). The ability to regenerate TE was not due to delay of development after 2inh. treatment, as the total numbers of cells in control E3.5 blastocysts (n = 15) and corresponding blastocysts cultured in 2inh. conditions (n = 15) were similar (62.9 ± 13.7 *vs*. 69.6 ± 7.2, respectively; p > 0.05).

Notably, in contrast to 32-cell stage (E3.0) ICMs, which regenerated the TE layer during the first 24 hrs of *in vitro* culture, E3.5 ICMs treated with 2inh. from 8-cell stage required 48 hrs of *in vitro* culture to form the TE layer *de novo*. After 24 hrs, despite initiating polarisation of outer cells, as confirmed by the apically localised phosphorylated ezrin/radixin/moesin (pERM) complex (n = 8; Fig. 5I and J), Cdx2 was not detected (4/9 embryos, 44%) or a weak signal of this protein was present only in single outer cells (5/9 embryos, 56%). To investigate further whether TE regeneration is a result of Hippo signalling inactivation^37^, we analysed E3.5 ICMs, originating from embryos pre-incubated in 2inh. conditions from the 8-cell stage, after 24 hrs of culture for the presence of Hippo pathway component – phospho-Yes-associated protein (pYAP) and nuclear YAP. We found that in freshly isolated ICMs pYAP was localised in the cytoplasm of all cells (n = 9 ICMs; Fig. 5K), whereas 24 hrs after isolation by IS ICMs contained outer cells devoid of pYAP (n = 12 ICMs; Fig. 5L). To confirm the involvement of Hippo pathway in the initiation of *Cdx2* expression and, consequently, TE regeneration, we immunostained nuclear YAP in ICMs treated previously with 2inh. Consistently with the results for pYAP, immediately after immunosurgery all ICMs lacked nuclear YAP (n = 8 ICMs; Fig. 5M), while after 24 hrs of *in vitro* culture localisation of nuclear YAP was detected in most of outer cells (n = 16 ICMs; Fig. 5N).

These data show that blocking FGF/MAPK signalling in isolated ICMs prevents the transition from totipotency to lineage priming, i.e. extends the window during which ICM cells can differentiate into TE. TE regeneration results from polarity-induced inactivation of Hippo signalling in outer cells of isolated ICMs.

## Discussion

The blastocyst stage in mouse lasts from E3.0 32-cell embryo with a nascent cavity to an expanded blastocyst with more than 140 cells, able to implant in the uterus on E4.5. Despite many years of research, the exact moment at which ICM cells of the blastocyst definitely lose their totipotency (defined as the ability to differentiate into all primary cell lineages) and the mechanism responsible for the switch from totipotency to lineage priming still remains unknown.

We demonstrated that only E3.0 ICMs (isolated from 32-cell stage blastocysts) are able to regenerate TE. This ability disappears at E3.5 (50–70 cell) stage. It means that despite the spatial segregation of ICM and TE, ICM cells of early blastocysts remain totipotent. This accords with our previous results, where we showed that aggregates of exclusively inner cells of disaggregated 32-cell blastocysts initiate *Cdx2* expression and reform blastocysts with ICM and TE^38^. Our findings are also consistent with the results regarding multilineage differentiation potency of isolated E3.0 ICMs, published in the 1970s^39,40^. According to some authors, this ability is preserved longer in development, until E3.5 ICMs^41–43^. There are two possible explanations of the discrepancies between these results. Firstly, as most of the authors staged the embryos based on the days *post coitum* or on the size of the cavity rather than the cell number, the blastocysts used in experiments could have significantly differed in respect to the developmental stage at the time of the ICM isolation. Secondly, methods of the ICM isolation do not guarantee the complete removal of TE cells, leading to a contamination of the ICMs with non-lysed TE cells. Indeed, Szczepanska *et al*. showed that in ICMs derived from E3.5 blastocysts composed of approximately 50–70 cells TE does regenerate, however, it originates from the TE cells that avoided IS-induced lysis^44^. In contrast to Szczepanska and collaborators, we did not notice TE regeneration in E3.5 ICMs, probably due to higher effectiveness of our TE removal method.

The ability to regenerate TE on the surface of ICM seems to be related to the period when *Cdx2* expression overlaps with other lineage-specific transcription factors (until 32-cell stage in mouse)^45,46^. It has been also shown that until early (approximately 35-cell) blastocyst stage there are some Cdx2-positive cells, which can translocate from the outer position inward, down-regulate *Cdx2* expression, probably by the activation of Hippo signalling, and finally contribute to the ICM^47,48^. However, considering the low frequency of such events, it does not seem plausible that these cells alone, after TE removal and exposure to the outside environment, are sufficient to regenerate TE layer. On the other hand, studies on Hippo signalling pathway demonstrated that ICM cells exposed to external environment after IS establish apical-basolateral polarity, which in turn leads to the suppression of Hippo pathway and changes in subcellular localisation of YAP. Relocation of YAP to the nucleus in these cells allows for the initiation of *Cdx2* expression and change of cell fate into TE^28^. However, it was also shown that ICMs isolated from *Cdx2*-null 32-cell stage embryos, similarly to wild-type ICMs, have the ability to regenerate a polarised epithelial TE-like layer^37^, indicating the impact of additional or distinct mechanisms that determine this capacity and control cell fate. Our observation that loss of the capacity of TE regeneration coincided with the emergence of PE and EPI precursors within the ICM led us to hypothesize that FGF/MAPK signalling may be responsible for the restriction of ICM plasticity. Indeed, we showed that the inhibition of FGF/MAPK signalling maintains the ability of E3.5 ICMs to regenerate TE, as a result of polarity-induced Hippo inactivation. We positively verified this thesis by showing lack of pYAP signal in the cytoplasm and the presence of nuclear YAP in outer ICM cells after 24 hrs of culture, which proves dephosphorylation of the protein and its shuttling to the nuclei of these cells. It is known that nuclear accumulation enables interaction of YAP with the transcription factor Tead4 and in consequence promotion of TE and suppression of ICM developmental program^28^.

It has been shown using *Fgf* mutant embryos, that FGF signalling is required for the establishment of PE precursors at the 64-cell stage by maintenance of *Gata6* within the cells of the ICM^8^. Potentially a reciprocal inhibition between PE- and TE-specific transcription factors could restrict plasticity of the ICM cells. Indeed, it has been demonstrated that in the absence of *Gata6* all ICM cells acquire EPI identity^11,49^. However, there may be rare exceptions: some cells of Gata6^−/−^ ICMs were identified as double-negative for Nanog/Gata6 and expressed *Cdx2* instead^11^. Therefore, it seems possible that ICMs, which are devoid of TE and need to create epithelium on their surface, in the absence of FGF/MAPK signalling, and in consequence - failing to maintain Gata6 activity – up-regulate TE markers, such as *Cdx2*, as a result of polarity-induced Hippo inactivation, finally acquiring the TE fate. The molecular cross-talk between these two regulatory pathways needs further investigation.

Our data revealed that the potency of ICM cells derived from E3.0 blastocyst varies largely depending on their environment. It appears that at the E3.0 blastocyst stage ICM cells exhibit “hidden” flexibility, which is manifested by multi-directional (towards TE, PE and EPI) differentiation, when they are out of their natural milieu. Rossant and Lis showed that ICMs of 30–60-cell blastocysts when combined with morulae occasionally contributed not only to the ICM- but also to the TE-derived tissues of the chimaeric postimplantation conceptuses^43,50^. This plasticity has been confirmed by Grabarek *et al*., who combined ICM cells derived from subsequent stages of blastocysts (32–170-cell) with 8-cell embryos and tracked their fate in resulting chimaeras. Our experiments additionally indicate that progeny of 32-cell stage blastomeres contribute to TE, EPI or PE with different efficiency depending on the host embryo environment. The significance of the “niche” was also shown by Posfai *et al*.^51^. Using mouse line expressing fused Cdx2-eGFP protein they showed that when cells exhibiting low levels of Cdx2-eGFP protein (presumptive ICM cells) are aggregated with 8-cell embryos, they contribute exclusively to the ICM already at the early 32-cell stage. However, when re-constructed into homogeneous (inner cells-only or outer cells-only) aggregates, they lose their potential to make TE as late as by 64-cell E3.5 blastocyst stage. Authors suggested that the morula aggregation assay reveals the ability to specificate into particular lineage but not necessarily the whole developmental potential of these cells, which is revealed in the re-aggregation experiments^51^.

Plasticity of ICM cells, in regard to their differentiation either to EPI or to PE, is maintained until around E4.5. Grabarek *et al*. revealed that even at the late blastocyst stage (<120 cells) PE and EPI cells maintain the capacity to switch between alternative fates in the chimaeric embryos, albeit PE cells retain this plasticity longer than EPI cells^52^. Results of our experiments using live imaging of isolated ICMs also prove that at this stage some PE and EPI cells maintain plasticity, despite the expression of lineage-specific markers, and still can mutually switch their fates (i.e. EPI to PE, and vice versa). Whether this ability is utilised in undisturbed embryos, which, in contrast to the isolated ICMs are surrounded by the TE epithelium, is questionable. Experimental intervention might trigger a response, i.e. the need to cover the whole surface with PE cells, which otherwise would not be a part of normal developmental program. However, ICM cells down-regulating *Pdgfrα* ^*H2B-GFP*^ and acquiring the EPI fate were observed in intact embryos as late as beyond the 128-cell stage^7^. Occasional switch from PE to EPI fate was also noticed in embryos expressing a *Nanog* reporter^53^. On the other hand, since we followed only single-lineage live imaging reporter we cannot exclude that *Pdgfrα* ^*H2B-GFP*^-expressing cells were in fact bipotential cells, co-expressing markers of both PE and EPI. Saiz *et al*. showed that only such cells, which persist in blastocysts of up to 120 cells, may choose between alternative fates due to ability to respond to FGF signalling modulation^33^. Overall, all these results argue that this asynchronous allocation mechanism may operate in normal development, to ensure the balanced numbers of cells of respective lineages within the blastocyst.

Using chimaera assay it was shown that the complete loss of ICM cell plasticity and final commitment to one of the three cell lineages (TE, EPI or PE) occurs between 120- and 170-cell blastocyst stage^52^. Regardless of the host embryo environment, PE and EPI cells at this stage are developmentally committed, since after microinjection of dissociated ICM cells into recipient 8-cell embryo or blastocyst their contribution in resulting chimaeras is restricted exclusively to the lineage of their origin^52,54^.

It is worth noting that our results explain the correlation between the stage of blastocysts serving as a source of ICMs and the efficiency of ESC derivation, which has been reported previously both in mouse and human^22,25^. ESCs can be derived from any preimplantation stage, albeit with different frequency^22^ even following FGF/MAPK inhibition, which generally facilitates the isolation of germline-competent ESCs^10^. Our data point the ICM of about E3.5 blastocyst as the most optimal developmental stage for ESC derivation. Indeed, Boroviak *et al*. showed that in contrast to this mid-blastocyst stage, single ICM cells of early blastocysts (about 32-cell) when cultured in 2i conditions hardly ever generated ESCs^22^_,_ giving rise to large and vacuolated cells displaying key TE markers. Thus, the 2inh.-mediated full potency of early ICMs, observed in our study, may underlie the inability to obtain ESC lines from this stage.

## Methods

All animal studies were approved by the Local Ethics Committee for Experimentation on Animals no. 1 (Warsaw, Poland), designated by the National Ethics Committee for Experimentation on Animals (Poland), and were performed in compliance with the national regulations.

### Animals

F1(C57Bl6/TarxCBA/Tar) or CD females and F1(C57Bl6/TarxCBA/Tar) and Pdgfrα^H2B-GFP34^ or C57Bl/6-Tg(UBC-GFP)30Scha/J males were used in this study.

### Embryos

Blastocysts were obtained from F1 or CD females ovulating spontaneously or superovulated with 10 IU of PMSG (pregnant mare’s serum gonadotropin; Intervet) followed after 48 hrs with 10 IU of hCG (human chorionic gonadotropin; Intervet), and mated with F1, Pdgfrα^H2B-GFP^ or C57Bl/6-Tg(UBC-GFP)30Scha/J males. To obtain E3.0 blastocysts, 8-cell embryos were isolated from oviducts of the F1 females caged with F1 or C57Bl/6-Tg(UBC-GFP)30Scha/J males and sacrificed 2 days after the vaginal plug had been found. The embryos were then cultured in KSOM medium (Specialty Media) for about 20 hrs and inspected for the appearance of the cavity. To obtain E3.5 and E4.5 blastocysts females with vaginal plugs were autopsied on the 4th day *post coitum* (*dpc*) or 92–96 hrs after hCG injection. Blastocysts were collected by flushing oviducts and uteri with M2 medium supplemented with 4 mg/ml bovine serum albumin (BSA; Sigma-Aldrich^55^).

Blastocysts in sequential stages of development were used:

- E3.0 (32-cell-stage) – isolated as described above;
- E3.5–3.75 (50–70-cell-stage) – obtained after 92–96 hrs post hCG or on 4 *dpc* in case of spontaneously ovulated females, and used for experiments immediately after isolation;
- E4.5 (>80-cell-stage) – obtained as E3.5 blastocysts and cultured 24 hrs *in vitro*.

*In vitro* culture of embryos was conducted in standard culture conditions (37.5 °C, in 5% CO_2_ in the air).

### Labelling of TE cells in E3.0 blastocysts with fluorescent latex microspheres

The *zona pellucida* was removed from E3.0 embryos using acidic Tyrode’s solution^56^. Embryos were washed in M2 medium (without BSA) and incubated with latex fluorescent microspheres (FM, Fluoresbrite plain YG0.2 mm Microspheres, Polysciences, Inc; 1:100, 5 min^57^).

### Microinjection of inner blastomeres derived from E3.0 blastocysts into 8-cell embryos and blastocysts

Recipient embryos, i.e. 8-cell embryos and E3.5 blastocysts, were collected from F1 females mated with F1 males. They were obtained 64 and 94 hrs after hCG injection, respectively.

Donor 32-cell blastocysts were obtained by *in vitro* culture of 8-cell embryos isolated from F1 females mated with C57Bl/6-Tg(UBC-GFP)30Scha/J males. Single cells derived from 32-cell embryos were obtained as previously described^38^. In brief, embryos were labelled with multifluorescent microspheres (FM, Fluoresbrite Multifluorescent 0.2 micron Microspheres; Polysciences, Inc; 1:100 in M2 without BSA, 5 min^57,58^) to label outer cells, pre-incubated in pronase (0.5%, 5 min; Calbiochem) followed by incubation in Ca^2+^- and Mg^2+^-free M2 medium with EGTA (0,2 mg/ml; 30 min; Sigma-Aldrich), and disaggregated by pipetting into single cells. Inner cells were separated from outer cells based on the presence (outer cells) and absence (inner cells) of FM.

#### Microinjection

Inner blastomeres were introduced into the 8-cell embryos (de-compacted by 10 min treatment with Ca^2+^- and Mg^2+^-free M2 medium with EGTA) or E3.5 blastocysts under an inverted microscope (Nikon Eclipse TS100) with the aid of piezoelectric micromanipulator (PM-10-1, Martzhauser, Wetzlar-Steindorf). Injection pipette (diameter: 28 µm) was prepared using P-97 puller (Flaming/Brown micropipette puller, Sutter Instrument Co.). Four inner cells were introduced into the cavity of blastocysts and 1 inner cell was injected into 8-cell embryos. Blastocysts and 8-cell embryos were cultured for 48 and 72 hrs, respectively, in KSOM.

### Labelling of PE cells of E4.5 blastocysts with lipophilic fluorescent DiI dye

DiI dye (Vybrant®, 1:100; Life Technologies) was injected into the cavity of blastocyst with the piezoelectric micromanipulator to label the cells relining the cavity, i.e. PE and mural TE.

### Immunosurgery

Blastocysts were incubated in anti-mouse serum followed by incubation in the guinea pig complement (both 1:3, 30 min, Sigma-Aldrich)^59^. Lysed trophectoderm cells were removed by pipetting.

### Treatment of embryos and ICMs with FGF/MAPK inhibitors

The 8-cell embryos and isolated E3.5 ICMs were treated with a combination of a MEK kinase inhibitor (PD0325901, 500 nM, Stemgent)^10,12^ and a FGF receptor inhibitor (PD173074, 100 nM, Stemgent) in KSOM medium (henceforth referred to as 2inh. conditions)^12^.

### Immunostaining

Blastocysts and ICMs were fixed in 4% paraformaldehyde (30 min, Biomed), permeabilized in PBS containing 0.5% Triton X-100 (30 min, RT, Sigma-Aldrich) and blocked in PBS with 3% BSA and 0.05% Tween20 (overnight in 4 °C; Bio-Rad Laboratories). Primary antibodies used were: rabbit polyclonal antibody against Gata4 (1:100, Santa Cruz, #sc-9053), goat polyclonal antibody against Sox17 (1:100, R&D Systems, #AF1924), mouse monoclonal antibody against Oct4 (1:100, Santa Cruz, #sc-5279), rabbit polyclonal antibody against Nanog (1:100, ReproCELL, #RCAB002P-F), rabbit polyclonal antibody against Sox2 (1:100, Abcam, #ab97959), mouse monoclonal antibody against Cdx2 (1:50, BioGenex, #MU392A-UC), mouse monoclonal antibody against YAP1 (1:100, Abcam, #ab56701), rabbit polyclonal antibody against pYAP (1:100, Cell Signaling Technology, #4911), rabbit monoclonal antibody against pERM (1:400, Cell Signaling Technology, #3141). After 24 hrs of incubation in 4 °C the embryos and ICMs were rinsed with PBS and incubated with the mixture of corresponding secondary antibodies: Alexa 647-conjugated donkey anti-rabbit (1:200, Invitrogen, #A31573), Alexa 488-conjugated donkey anti-goat IgG (1:200, Invitrogen, #A11055), rhodamin-conjugated donkey anti-mouse IgG (1:200, Jackson Immunoresearch Laboratories, #115-025-068) or Alexa 594-conjugated donkey anti-mouse IgG (1:200; Invitrogen, #A21203) for 2 hrs in RT and rinsed again. Antibodies were diluted in PBS with 3% BSA and 0.05% Tween20 (Sigma-Aldrich). DNA was labelled (15 min incubation, 37, 5 °C) with Draq5 dye (10 μM; Biostatus Ltd.) or chromomycin A_3_ (0.01 mg/ml, Sigma-Aldrich). Confocal images were acquired with LSM 510 Zeiss inverted confocal microscope (Jena, Germany) or Nikon A1R inverted confocal microscope (Nikon Instruments). LSM Image Browser or Imaris (Bitplane) were used to analyze the images. Optical sections were taken every 3.5 µm through fixed embryos and every 2.5 µm through ICMs. The number of nuclei in embryos was counted and the correlation between the presence or absence of GFP/DiI marker and Gata4 was recorded.

### Time-lapse imaging

Time-lapse imaging of ICMs and embryos was performed on confocal microscope (Nikon A1R – A1 Confocal, Nikon Instruments Inc.) equipped with Okolab incubation chamber. Two-channel (green fluorescence to follow cells expressing histone H2B tagged with GFP under the control of Pdgfrα promoter and transmitted light to visualize whole embryos) images were acquired every 20 min, every 2.5 μm. Imaris software (Bitplane) was used for cell tracking.

### Statistical analysis

Quantitative data was shown as mean ± standard deviation (SD), t-Student Test was used for the statistical analysis; p < 0.05 was considered statistically significant. For comparison of the origin of the ultimate PE and the fate of *Pdgfrα* ^*H2B-GFP*^-expressing cells we used t-Student Test performed based on the fractions, implied as quotient of cell number in one of the 4 groups and number of all cells either building the ultimate PE or all Pdgfrα(+) cells, respectively.

## Electronic supplementary material

Supplementary information

Supplementary movie 1

Supplementary movie 2
