# Supplementary material for: Plasticity of the inner cell mass in mouse blastocyst is restricted by the activity of FGF/MAPK pathway
Source: Sci Rep. 2017 Nov 9;7:15136. doi: 10.1038/s41598-017-15427-0 (PMC5680175; doi:10.1038/s41598-017-15427-0)
Supplement: Supplementary file 1 — Supplementary information [file 41598_2017_15427_MOESM1_ESM.pdf]

Plasticity of the inner cell mass in mouse blastocyst is restricted by the activity of FGF/MAPK pathway

Wigger M.[#1], Świtoń K.[#1,2], Filimonow K.[#1,2,§], Plusa B.[2], Maleszewski M.[1], Suwińska A.*[1]

[1] Department of Embryology, Faculty of Biology, University of Warsaw, Warsaw, Poland

[2] Faculty of Life Sciences, University of Manchester, Manchester, UK

[#] these authors contributed equally to this work

[§] Current address: Department of Experimental Embryology, Institute of Genetics and Animal Breeding, Polish Academy of Sciences, Jastrzębiec, Poland

* Corresponding author: Aneta Suwińska

Fax: 48 22 5541210

E-mail address: asuwinska@biol.uw.edu.pl

Address for correspondence: Department of Embryology, Faculty of Biology, University of Warsaw, Miecznikowa 1, 02-096 Warsaw, Poland

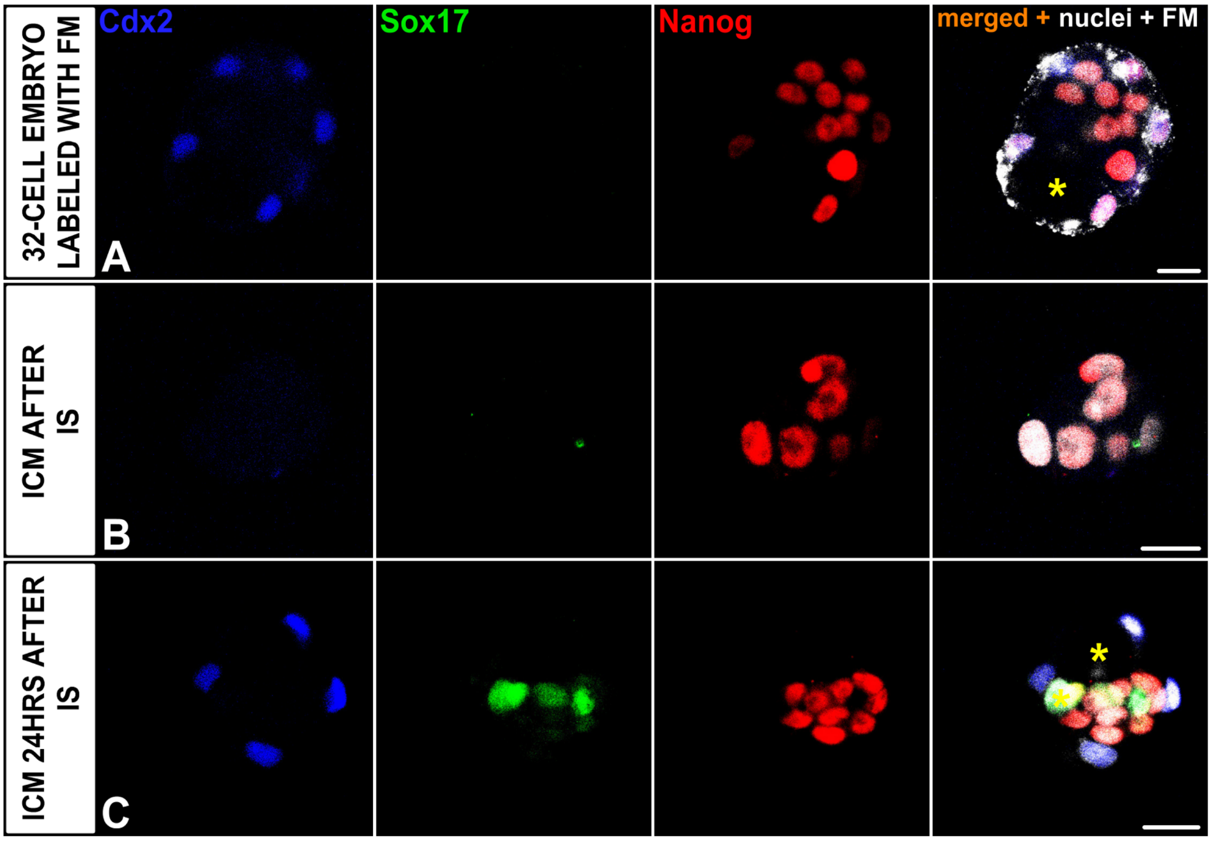

Figure S1. Control 32-cell blastocysts and their ICMs

(A) E3.0 blastocyst labelled with the fluorescent microspheres (FM), **(B)** E3.0 ICM immediately after isolation, **(C)** E3.0 ICM 24 hrs after IS. Blue: Cdx2, green: Sox17, red: Nanog, white: nuclei and FM; right panel shows merged pictures; yellow (*) indicates blastocyst cavity. Scale: 20µm.

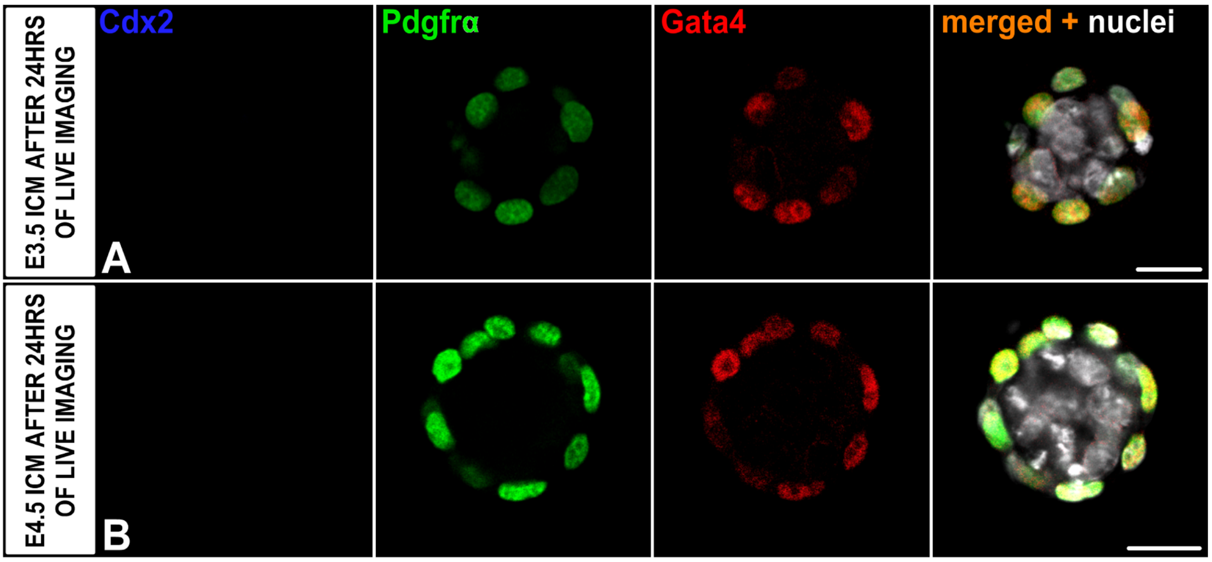

Figure S2. E3.5 and E4.5 ICMs subjected to live imaging.

(A) E3.5 ICM and **(B)** E4.5 ICM after 24 hrs of live imaging, Blue: Cdx2, green: Pdgfrα$^{H2B\text{-}GFP}$, red: Gata4, white: nuclei; right panel shows merged pictures. Scale: 20µm.

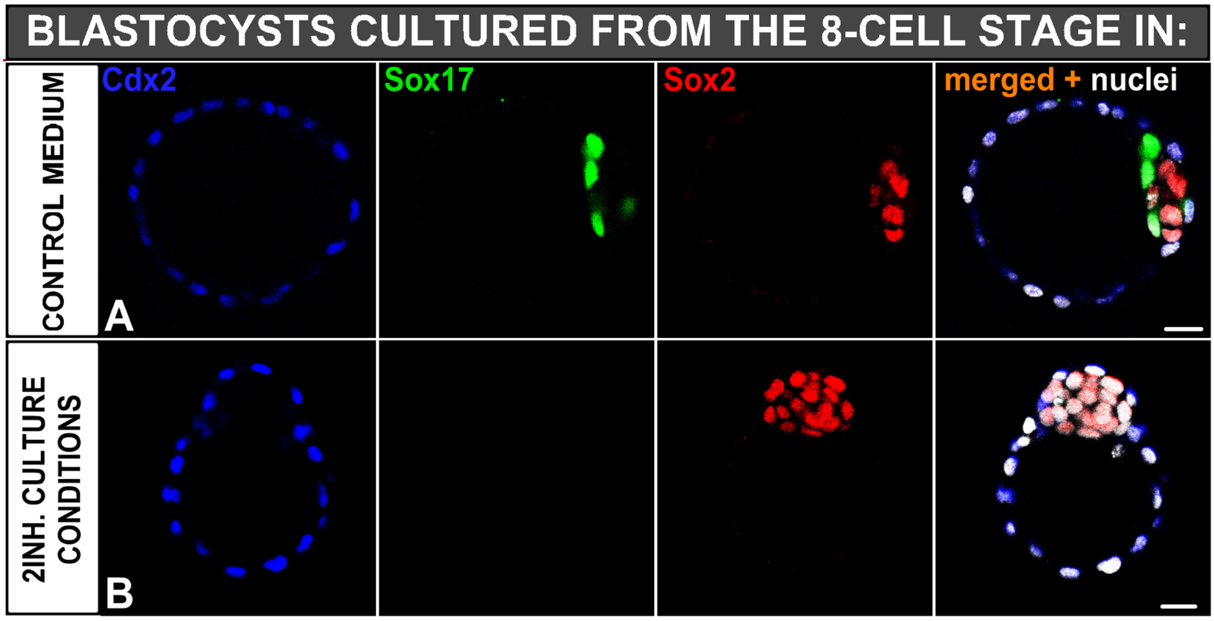

Figure S3. Effect of FGF/MAPK inhibition on blastocysts.

(A) Blastocyst cultured from the 8-cell stage in control medium and **(B)** in 2inh. conditions. Blue: Cdx2, green: Sox17, red: Sox2, white: nuclei; right panel shows merged pictures. Scale: 20µm.

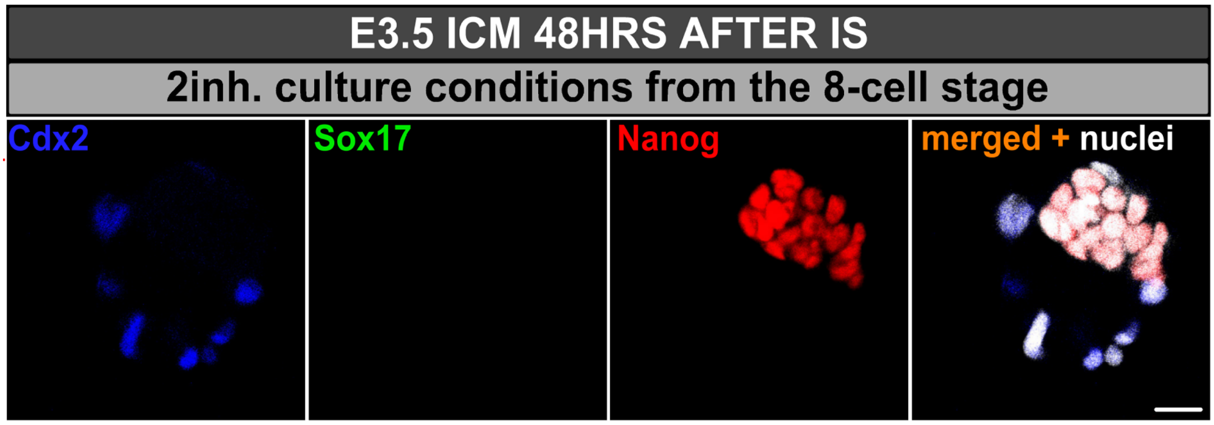

Figure S4. Effect of FGF/MAPK inhibition on isolated E3.5 ICMs

ICMs were derived from E3.5 blastocysts pre-incubated in 2inh. media from the 8-cell stage. Blue: Cdx2, green: Sox17, red: Nanog, white: nuclei; right panel shows merged pictures. Scale: 20µm.

Movie 1. Time-lapse imaging of E3.5 ICM

Movie 2. Time-lapse imaging of E4.5 ICM

Table S1. The source of the ultimate PE in individual E3.5 and E4.5 ICMs subjected to time-lapse imaging.

No of ICM	Pdgfrα$^{(+)}$ cells localised on the surface of ICM from the beginning of culture	Pdgfrα$^{(+)}$ cells, which were localised inside the ICM and translocated to the surface	Pdgfrα$^{(-)}$ cells, which were localised inside the ICM, but up-regulated *Pdgfra* and migrated to the surface	Pdgfrα$^{(-)}$ cells up-regulating *Pdgfra* while localised on the surface of ICM
**E3.5 ICMs**				
1	1	0	1	10
2	4	0	1	7
3	4	3	0	0
4	5	1	0	0
5	3	1	4	4
6	5	0	1	0
7	2	1	0	2
8	2	0	7	2
9	5	1	1	1
10	4	1	0	1
11	5	0	1	7
12	1	0	0	5
13	3	0	4	6
14	7	0	3	4
15	5	0	0	0
16	4	0	1	2
17	5	0	0	0
**E4.5 ICMs**				
1	11	0	0	1
2	9	0	0	2
3	3	0	0	0
4	14	0	0	2
5	13	0	0	0
6	14	0	0	0
7	7	1	0	1
8	19	0	0	0
9	11	0	0	1

Table S2. The fate of Pdgfrα(+) cells in individual E3.5 and E4.5 ICMs subjected to time-lapse imaging.

No of ICM	Pdgfrα(+) cells localised on the surface of ICM from the beginning of culture	Pdgfrα(+) cells, which were localised inside the ICM and translocated to the surface	Pdgfrα(+) cells down-regulating *Pdgfra* while localised inside the ICM	Pdgfrα(+) cells, which underwent cell death
**E3.5 ICMs**				
1	1	0	1	1
2	4	0	1	3
3	4	3	0	4
4	5	1	2	1
5	3	1	0	3
6	5	0	2	1
7	2	1	0	1
8	2	0	1	2
9	5	1	0	2
10	4	1	0	1
11	5	0	0	3
12	1	0	0	1
13	3	0	0	2
14	7	0	0	4
15	5	0	0	2
16	4	0	0	2
17	5	0	0	2
**E4.5 ICMs**				
1	11	0	5	5
2	9	0	0	10
3	3	0	0	5
4	14	0	1	1
5	13	0	0	2
6	14	0	0	1
7	7	1	3	7
8	19	0	0	1
9	11	0	4	7
